# Characterisation of the oxysterol metabolising enzyme pathway in mismatch repair proficient and deficient colorectal cancer

**DOI:** 10.18632/oncotarget.10224

**Published:** 2016-06-22

**Authors:** Rebecca Swan, Abdo Alnabulsi, Beatriz Cash, Ayham Alnabulsi, Graeme I. Murray

**Affiliations:** ^1^ Pathology, School of Medicine, Medical Sciences and Nutrition, University of Aberdeen, Aberdeen, AB25, 2ZD, UK; ^2^ Vertebrate Antibodies Ltd, Zoology Department, University of Aberdeen, Aberdeen, AB24 2TZ, UK

**Keywords:** biomarker, colorectal cancer, cytochrome P450, oxysterol, prognosis

## Abstract

Oxysterols are oxidised derivatives of cholesterol, formed by the enzymatic activity of several cytochrome P450 enzymes and tumour-derived oxysterols have been implicated in tumour growth and survival. The aim of this study was to profile the expression of oxysterol metabolising enzymes in primary colorectal cancer and assess the association between expression and prognosis.

Immunohistochemistry was performed on a colorectal cancer tissue microarray containing 650 primary colorectal cancers using monoclonal antibodies to CYP2R1, CYP7B1, CYP8B1, CYP27A1, CYP39A1, CYP46A1 and CYP51A1, which we have developed. Unsupervised hierarchical cluster analysis was used to examine the overall relationship of oxysterol metabolising enzyme expression with outcome and based on this identify an oxysterol metabolising enzyme signature associated with prognosis.

Cluster analysis of the whole patient cohort identified a good prognosis group (mean survival=146 months 95% CI 127-165 months) that had a significantly better survival (δ^2^=12.984, p<0.001, HR=1.983, 95% CI 1.341-2.799) than the poor prognosis group (mean survival=107 months, 95% CI 98-123 months). For the mismatch repair proficient cohort, the good prognosis group had a significantly better survival (δ^2^=8.985, p=0.003, HR=1.845, 95% CI 1.227-2.774) than the poor prognosis group. Multi-variate analysis showed that cluster group was independently prognostically significant in both the whole patient cohort (p=0.02, HR=1.554, 95% CI 1.072-2.252) and the mismatch repair proficient group (p=0.04, HR=1.530, 95% CI 1.014-2.310).

Individual oxysterol metabolising enzymes are overexpressed in colorectal cancer and an oxysterol metabolising enzyme expression profile associated with prognosis has been identified in the whole patient cohort and in mismatch repair proficient colorectal cancers.

## INTRODUCTION

Colorectal cancer is one of the most common types of malignancy affecting both men and women, with a worldwide annual incidence of greater than 1.2 million new cases [1, 2]. The disease remains a leading cause of cancer-related mortality and, despite gradual improvements in prognosis, the 5-year survival remains relatively poor at approximately 55% [1]. Colorectal cancer develops slowly over several years and symptoms often only become apparent in the late stages, therefore many colorectal cancers present at an advanced stage. Patients presenting with distant metastatic disease have a 5-year survival of less than 10% [1].

Currently, colorectal cancer is commonly staged using the tumour, node, metastasis (TNM) staging system to guide treatment decisions and indicate prognosis. However, patients with the same stage of tumour often experience a wide range of different clinical outcomes. Despite the unequivocal value of current staging systems, there is a still need to develop reliable biomarkers to more accurately predict prognosis and risk stratify patients with colorectal cancer. Biomarkers can have a variety of roles in colorectal cancer including early detection, predicting prognosis, predicting response to therapy and aiding post-operative monitoring [3].

Oxysterols are oxidised derivatives of cholesterol, formed by the enzymatic activity of several cytochrome P450 enzymes [4, 5]. Oxysterols function as key signalling molecules involved in the development and functioning of the immune system and the maintenance of cellular cholesterol homeostasis [6–12]. In addition to the established role of oxysterols in normal immune system functioning, it is increasingly acknowledged that the oxysterol pathway plays a role in tumourigenesis through altering host anti-tumour immunity. For example, oxysterols have been demonstrated to down-regulate the G-protein coupled receptor chemokine receptor 7 (CCR7) through activation of the ligand-activated transcription factor LXRα in dendritic cells [13]. CCR7 is involved in the migration of dendritic cells to draining lymph nodes, thus suppression of this chemokine receptor results in trapping of dendritic cells in the tumour and subsequent interference with antigen presentation to anti-tumour T-cells [7]. Through suppression of CCR7 in an LXR-dependent manner, oxysterols impede host anti-tumour immunity. A further mechanism whereby oxysterols may promote tumour progression is via chemo-attraction of neutrophils [14, 15]. Invading neutrophils may provide a critical growth and survival advantage in many solid tumours due to production of the pro-angiogenic factors prokineticin-2 and matrix metalloproteinase-9 [16].

Despite the recognition of the role of oxysterol signalling in tumourigenesis, the key cytochrome P450s involved in the oxysterol pathway have received very limited study in existing research with regard to their expression in tumours [17, 18]. This study has profiled the expression of the cholesterol metabolising enzymes CYP2R1, CYP7B1, CYP8B1, CYP27A1, CYP39A1, CYP46A1 and CYP51A1 in primary colorectal cancer tissue using a well-characterised cohorts of colorectal cancers. The clinico-pathological significance of each of the cytochrome P450s studied was determined, including the relationship between expression and overall survival. An oxysterol metabolising enzyme expression profile associated with prognosis has been identified in the whole patient cohort and in mismatch repair proficient colorectal cancers.

## RESULTS

### Monoclonal antibodies to oxysterol metabolising enzymes

**Figure 1:**
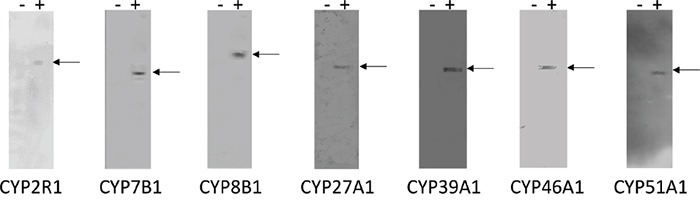
Immunoblots of CYP2R1, CYP7B1, CYP8B1, CYP27A1, CYP39A1, CYP46A1 and CYP51A1 monoclonal antibodies The left hand lane (−) of each panel contains control cell lysate while the right hand lane (+) of each panel contains lysate prepared from cells over expressing the relevant protein. Fifteen micrograms of protein were loaded per well except CYP51A1 (five micrograms of protein). No bands were detected with the control lysate, while a band migrating at the expected molecular weight was observed for each antibody in the lane containing the relevant expressed protein.

The specificity of the monoclonal antibodies to CYP2R1, CYP7B1, CYP8B1, CYP27A1, CYP39A1, CYP46A1 and CYP51A1 was determined by ELISA using the immunogenic peptides and also by immunoblotting using whole cell lysates from cells overexpressing of each protein (Figure 1). A band migrating at the expected molecular weight was observed for each antibody in a lysate prepared from cells overexpressing the relevant protein while no bands were detected with the corresponding control lysate.

### Expression of oxysterol metabolising enzymes in primary and metastatic colorectal cancer

**Figure 2:**
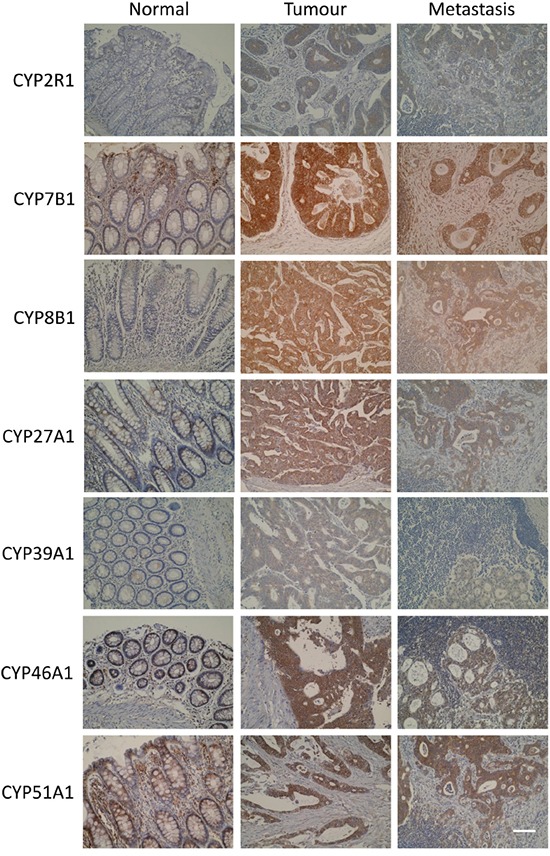
Photomicrographs of CYP2R1, CYP7B1, CYP8B1, CYP27A1, CYP39A1, CYP46A1 and CYP51A1 in normal colonic mucosa, primary colorectal cancer and metastatic colorectal cancer (original magnification x 300, scale bar represents 100μm)

Each antibody showed immunoreactivity in normal colonic epithelium, primary colorectal cancer and metastatic colorectal cancer. Where immunoreactivity was observed, immunostaining was localised to the tumour cell cytoplasm (Figure 2). Nuclear or membranous staining was not observed. Whole section immunohistochemistry of a sub-set of tumours showed no evidence of intra-tumour heterogeneity of expression of any of the oxysterol metabolising enzymes.

**Figure 3:**
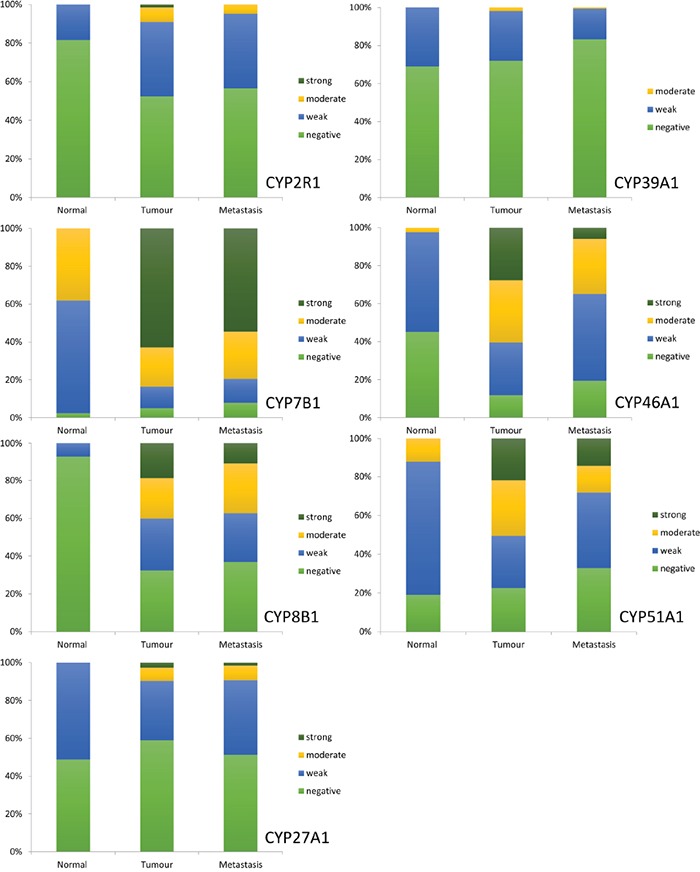
The frequency distribution of the intensity of expression of CYP2R1, CYP7B1, CYP8B1, CYP27A1, CYP39A1, CYP46A1 and CYP51A1 in normal colonic mucosa, primary colorectal cancer and lymph node metastasis

A general trend of increased expression in primary tumour cells compared to normal colonic mucosa was observed for all proteins studied (Figure 3). Furthermore, expression of each oxysterol metabolising enzyme was slightly reduced in lymph node metastasis compared to primary tumour. Immunostaining for CYP7B1 in primary tumours showed the highest proportion of strong immunostaining, with 62.8% of primary colorectal cancers displaying strong immunoreactivity for this target protein. Immunostaining for CYP46A1 showed 27.7% of primary tumours were strongly stained. Strong immunostaining for CYP51A1 was demonstrated in 21.9% of tumours and CYP8B1 immunoreactivity was classified as strong in 18.9% of tumours. The remaining target proteins had very low frequencies of strong immunostaining and no tumour showed strong CYP39A1 immunoreactivity.

The intensity of immunostaining was significantly higher in primary colorectal cancer compared with normal colonic mucosa for CYP2R1 (p<0.001), CYP7B1 (p<0.001), CYP8B1 (p<0.001), CYP46A1 (p<0.001) and CYP51A1 (p=0.001). CYP27A1 and CYP39A1 showed no statistically significant difference in expression between primary tumour and normal colonic mucosa (Table 1).

**Table 1:** Comparison of the expression of each oxysterol metabolising protein in normal colonic mucosa, primary colorectal cancer and lymph node metastasis

	Immunoreactivity (p value, normal versus primary tumour)	Change in expression in tumour	Immunoreactivity (p value, primary tumour versus lymph node metastasis)	Change in expression in lymph node	Immunoreactivity (p value, paired primary Dukes C tumour versus lymph node metastasis)	Change in expression in lymph node
CYP2R1	**p<0.001**	↑	p=0.172	-	**p=0.034**	↓
CYP7B1	**p<0.001**	↑	**p=0.035**	↓	p=0.116	-
CYP8B1	**p<0.001**	↑	p=0.106	-	**p=0.002**	↓
CYP27A1	p=0.517	-	p=0.108	-	p=0.960	-
CYP39A1	p=0.714	-	**p=0.001**	↓	**p<0.001**	↓
CYP46A1	**p<0.001**	↑	**p<0.001**	↓	**p<0.001**	↓
CYP51A1	**p=0.001**	↑	**p<0.001**	↓	**p=0.001**	↓

Evaluation of normal colonic epithelium versus primary tumour samples for immunoreactivity (Mann-Whitney U test, ↑ = increased in tumour, ↓ = decreased in tumour, -= no change between tumour and normal) and evaluation of primary Dukes C colorectal tumour samples and their corresponding metastasis samples for immunoreactivity (Wilcoxon signed rank sum test, ↑= increased in lymph node metastasis, ↓ = decreased in lymph node metastasis, -= no change between primary and metastatic tumour). Significant values are highlighted in bold.

When examining the difference in expression between all cases of primary colorectal cancer and lymph node metastasis CYP7B1 (p=0.035), CYP39A1 (p=0.001), CYP46A1 (p<0.001) and CYP51A1 (p<0.001) each showed a significant decrease in immunoreactivity in lymph node metastasis compared to primary tumour. There was no statistically significant difference in expression of CYP7B1 or CYP27A1 between the paired cases of Dukes C (stage 3) colorectal cancer and their corresponding lymph node metastasis. However, CYP2R1 (p=0.034), CYP8B1 (p=0.002), CYP39A1 (p<0.001), CYP46A1 (p<0.001) and CYP51A1 (p=0.001) each demonstrated significantly reduced expression in paired lymph node metastasis compared to Dukes C (stage 3) colorectal cancer.

### Relationship of individual oxysterol metabolising enzymes with clinico-pathological parameters

The relationship between expression of each protein and relevant clinico-pathological parameters are summarised in Table 2. Expression of CYP2R1, CYP8B1, CYP27A1, CYP39A1 and CYP46A1 showed significant associations with Dukes stage. CYP39A1 and CYP46A1 were also significantly associated with tumour stage and lymph node stage. CYP7B1, CYP8B1, CYP39A1, CYP46A1 and CYP51A1 each displayed a statistically significant relationship with location of tumour in the colon versus the rectum. When the anatomical site of the tumour was stratified as proximal colon, distal colon or rectum, significant associations were found with expression of CYP8B1, CYP27A1 and CYP51A1. When investigating the relationship between expression of each protein and the presence of extramural venous invasion, CYP8B1, CYP27A1, CYP39A1 and CYP46A1 showed correlations with this pathological variable. In addition to extramural venous invasion, CYP27A1 was also associated with mismatch repair protein status. Expression of CYP51A1 was associated with tumour differentiation and mismatch repair protein status.

**Table 2:** The relationship between expression of each oxysterol metabolising enzyme and pathological parameters

	Screen detected (yes v no)		Tumour site (colon v rectum)		Tumour site (proximal colon v distal colon v rectum)		Tumour differentiation (well/moderate v poor)		EMVI (present or absent)		Mismatch repair protein status (proficient v deficient)		Tumour stage		Lymph node stage		Dukes stage	
	χ2	p-value	χ2	p-value	χ2	p-value	χ2	p-value	χ2	p-value	χ2	p-value	χ2	p-value	χ2	p-value	χ2	p-value
CYP2R1	1.564	0.668	0.690	0.876	10.940	0.090	3.492	0.322	2.186	0.535	5.296	0.151	21.225	**0.012**	10.061	0.122	13.142	**0.041**
CYP7B1	4.147	0.246	8.004	**0.046**	8.515	0.203	3.231	0.357	0.305	0.959	5.446	0.142	5.241	0.813	8.267	0.219	7.359	0.289
CYP8B1	1.111	0.774	19.712	**<0.001**	21.593	**0.001**	4.502	0.212	8.577	**0.035**	0.785	0.853	7.536	0.581	32.766	**<0.001**	29.844	**<0.001**
CYP27A1	3.207	0.361	4.189	0.242	21.394	**0.002**	2.866	0.413	16.318	**0.001**	10.009	**0.018**	14.085	0.119	11.048	0.087	13.555	**0.035**
CYP39A1	12.651	**0.002**	7.851	**0.020**	8.357	0.079	0.271	0.873	12.643	**0.002**	0.120	0.942	16.766	**0.010**	39.193	**<0.001**	32.463	**<0.001**
CYP46A1	2.139	0.544	8.657	**0.034**	12.308	0.055	1.378	0.711	7.964	**0.047**	4.664	0.198	19.288	**0.023**	16.707	**0.010**	29.304	**<0.001**
CYP51A1	5.565	0.135	14.043	**0.003**	16.604	**0.011**	8.678	**0.034**	1.282	0.733	9.751	**0.021**	20.433	**0.015**	9.702	0.138	7.795	0.254

Significant values are highlighted in bold.

### Unsupervised hierarchical cluster analysis and identification of prognostic signature

**Figure 4:**
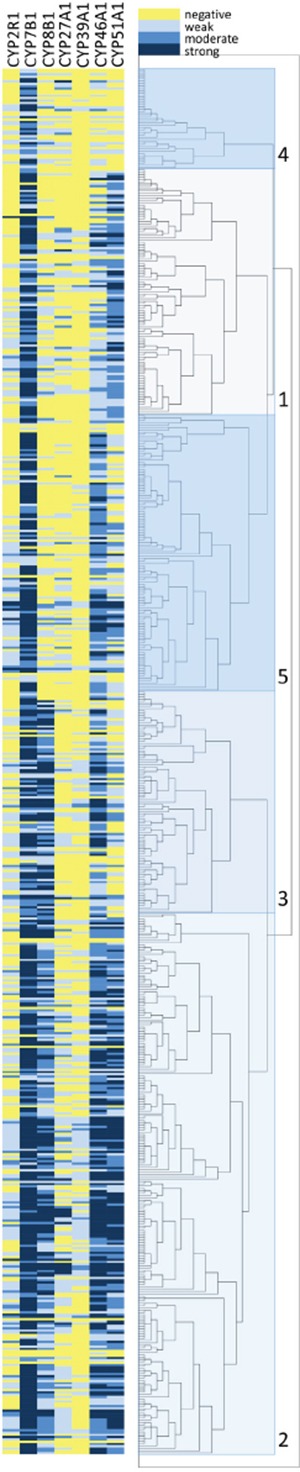
Unsupervised hierarchical cluster analysis of oxysterol metabolising enzymes Graphical representation of the immunohistochemistry marker data is shown in the left hand panel. The right hand panel shows the results of the hierarchical cluster analysis presented as a dendrogram with 5 individual clusters identified. Oxysterol metabolising enzymes are represented in columns and individual cases in rows.

**Figure 5:**
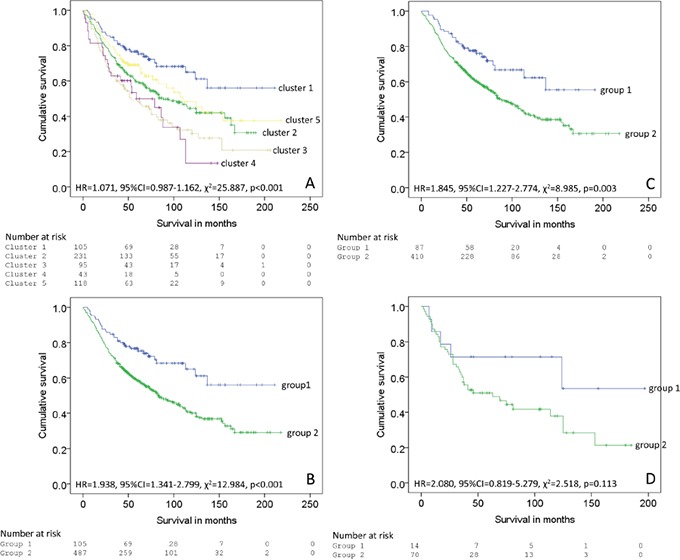
Survival analysis of individual clusters **A.** 5 clusters identified by hierarchical cluster analysis, **B.** The clusters classified into two groups (group 1=cluster 1 and group 2 = clusters 2-5 in the whole patient cohort, **C.** mismatch repair proficient cohort and **D.** mismatch repair deficient cohort

Unsupervised hierarchical cluster analysis was used as an unbiased exploratory statistical tool to examine the overall relationship of oxysterol metabolising enzyme expression with outcome and based on this identify an oxysterol metabolising enzyme signature associated with prognosis. A range of cluster solutions (number of clusters) was investigated to determine the optimum number of clusters that produced groups with different outcomes. Clustering the data into five clusters was identified as the optimum number of clusters for analysis in relation to the most prognostically significant groups (Figure 4). These five clusters were then combined into two groups; a good prognosis group (group 1, cluster 1) and a poor prognosis group (group 2, cluster 2-5) (Figures 4 and 5). The relationship of each cluster group and pathological parameters is shown in Table 3 and the expression of each oxysterol metabolising enzyme in each cluster group is shown in Table 4. The good prognosis group showed contained with low expression of CYP2R1 (p=0.002), CYP8B1 (p<0.001), CYP27A1 (p=0.028) and CYP46A1 (p<0.001) relative to the poor prognosis group.

**Table 3:** The relationship between cluster groups and pathological parameters in the whole patient cohort, mismatch repair proficient cohort and mismatch repair deficient cohort

	Screen detected (yes v no)		Tumour site (colon v rectum)		Tumour site (proximal colon v distal colon v rectum)		Tumour differentiation (well/moderate v poor)		EMVI (present or absent)		Mismatch repair protein status (proficient v deficient)		Tumour stage		Lymph node stage		Dukes stage	
Cluster group 1 v cluster group 2	χ2	p-value	χ2	p-value	χ2	p-value	χ2	p-value	χ2	p-value	χ2	p-value	χ2	p-value	χ2	p-value	χ2	p-value
Whole patient cohort	4.298	0.038	0.389	0.533	4.122	0.127	0.109	0.742	0.336	0.562	0.035	0.851	13.597	**0.004**	10.091	**0.006**	21.057	**p<0.001**
Mismatch repair proficient tumours	3.527	0.06	0.356	0.551	4.014	0.134	0.143	0.705	0.474	0.491	−	−	10.465	**0.015**	9.889	**0.007**	17.465	**p<0.001**
Mismatch repair deficient tumours	0.043	0.837	0	1	1.364	0.506	0.274	0.600	0	1	−	−	0.357	0.949	0.014	0.993	0.211	0.900

Significant values are highlighted in bold.

**Table 4:** The relationship between cluster groups and individual oxysterol metabolising enzymes in the whole patient cohort, mismatch repair proficient cohort and mismatch repair deficient cohort

	Whole patient cohort			Mismatch repair proficient cohort			Mismatch repair deficient cohort		
	χ^2^	p-value	Expression in cluster group 1 v group 2	χ^2^	p-value	Expression in cluster group 1 v group 2	χ^2^	p-value	Expression in cluster group 1 v group 2
CYP2R1	14.716	**0.002**	Low	9.847	**0.02**	Low	6.189	0.103	Low
CYP7B1	6.322	0.097	High	6.744	0.081	High	0.888	0.828	High
CYP8B1	75.659	**<0.001**	Low	56.049	**<0.001**	Low	19.408	**<0.001**	Low
CYP27A1	9.075	**0.028**	Low	6.316	0.097	Low	2.499	0.475	Low
CYP39A1	5.58	0.061	Low	6.844	**0.03**	Low	0.911	0.634	Low
CYP46A1	57.653	**<0.001**	Low	58.387	**<0.001**	Low	2.436	0.487	Low
CYP51A1	46.945	**<0.001**	High	40.233	**<0.001**	High	7.377	0.061	High

Significant values are highlighted in bold.

The good prognosis group (mean survival=146 months, 95% CI 127-165 months, n=105, number of deaths=32) had a significantly better survival (χ^2^=12.984, p<0.001, HR=1.983, 95% CI 1.341-2.799) than the poor prognosis group (mean survival=107 months, 95% CI 98-123 months, n=487, number of deaths=254) (Figure 5).

For the mismatch repair proficient cohort good prognosis group (mean survival=134 months, 95% CI 116-152 months, n=87, number of deaths=26) had a significantly better survival (χ^2^=8.985, p=0.003, HR=1.845, 95% CI 1.227-2.774) than the poor prognosis group (mean survival=110 months, 95% CI 107-126 months, n=410, number of deaths=206).

For the mismatch repair deficient cohort good prognosis group (mean survival=131 months, 95% CI 87-176 months, n=14, number of deaths=5) had a significantly better survival (χ^2^=2.518, p=0.113, HR=2.080, 95% CI 0.819-5.279) than the poor prognosis group (mean survival=84 months, 95% CI 76-113 months, n=70, number of deaths=42).

### Multi-variate analysis

Multi-variate analysis showed that cluster group was independently prognostically significant in both the whole patient cohort (p=0.02, HR=1.554, 95% CI 1.072-2.252) and the mismatch repair proficient group (p=0.04, HR=1.530, 95% CI 1.014-2.310) in a model that included information about Dukes stage (Table 5, Model A). Multi-variate analysis also showed that cluster group was independently prognostically significant in the whole patient cohort (p=0.033, HR=1.497, 95% CI 1.032-2.172) and not the mismatch repair proficient cohort in a model that contained tumour stage and lymph node stage (Table 6, Model B). Multi-variate analysis was also performed using only parameters that would be available at the time of a biopsy of colorectal cancer i.e. no pathological information about tumour stage, lymph node involvement or extramural venous invasion. In this case oxysterol metabolising enzyme signature was highly significant in both the whole patient cohort (p=0.002, HR=1.791, 95% CI 1.236-2.595) and the mismatch repair proficient group (p=0.011, HR=1.703, 95% CI, 1.129-2.568) (Supplementary Table 9).

**Table 5:** The significance of cluster group in multivariate analysis for the whole patient cohort and mismatch repair proficient cohort

Variable (categories)	Whole patient cohort			Mismatch repair proficient cohort		
	Wald value	p-value	Hazard ratio (95% CI)	Wald value	p-value	Hazard ratio (95% CI)
Age (< 70 v ≥ 70)	29.338	**<0.001**	1.976 (1.545-2.528)	23.283	**<0.001**	1.948 (1.486-2.553)
Tumour site (colon v rectum)	0.618	0.432	1.119 (0.846-1.481)	0.033	0.829	1.034 (0.764-1.3999)
EMVI (present v absent)	42.228	**<0.001**	2.366 (1.826-3.065)	36.257	**<0.001**	2.444 (1.827-3.268)
Dukes stage (A v B v C)	50.878	**<0.001**	0.350 (0.231-0.548)	23.030	**<0.001**	0.387 (0.25-0.645)
Cluster group (group 1 v group 2)	5.417	**0.02**	1.554 (1.072-2.252)	4.103	**0.043**	1.530 (1.014-2.310)

Model A includes Dukes stage as the parameter for assessing overall tumour stage.

Significant values are highlighted in bold.

**Table 6:** The significance of cluster group in multivariate analysis for the whole patient cohort and mismatch repair proficient cohort

Variable (categories)	Whole patient cohort			Mismatch repair proficient cohort		
	Wald value	p-value	Hazard ratio (95% CI)	Wald value	p-value	Hazard ratio (95% CI)
Age (< 70 v ≥ 70)	25.808	**<0.001**	1.901 (1.484-2.435)	20.958	**<0.001**	1.887 (1.438-2.477)
Tumour site (colon v rectum)	0.454	0.501	1.102 (0.831-1.462)	0.026	0.871	1.026 (0.756-1.392)
EMVI (present v absent)	24.777	**<0.001**	1.973 (1.510-2.579)	21.311	**<0.001**	2.038 (1.507-2.758)
Tumour stage (pT1 v pT2 v pT3 v pT4)	21.681	**<0.001**	0.363 (0.223-1.091)	14.770	**0.002**	0.569 (0.217-1.243)
Lymph node stage (pN0 v pN1 v pN2)	57.291	**<0.001**	0.296 (0.216-0.765)	34.912	**<0.001**	0.341 (0.238-0.826)
Cluster group (group 1 v group 2)	4.522	**0.033**	1.497 (1.032-2.172)	2.911	0.088	1.434 (0.948-2.170)

Model B include tumour (pT) stage and lymph node (pN) stage as the parameters for assessing overall tumour stage.

Significant values are highlighted in bold.

### The relationship of individual oxysterol metabolising enzymes, clinico-pathological parameters and survival

The relationship between expression of each protein and overall survival was investigated using different cut-off points of immunostaining intensity to allow a total of four comparisons to be made (negative v weak v moderate v strong, negative v positive, negative/weak v moderate/strong and negative/weak/moderate v strong). Supplementary Tables 2-8 details the association between each oxysterol metabolising enzyme and overall patient survival, using each comparison of immunoreactivity. Supplementary Figures 1-3 show the relationship of survival and individual oxysterol metabolising enzymes in the whole patient cohort, mismatch repair proficient tumours and mismatch repair deficient tumours.

Expression of CYP8B1 was consistently associated with patient survival. Considering each CYP8B1 intensity as a distinct group, increasing intensity was related to poorer prognosis (HR=1.191, 95% CI=1.074-1.32, χ^2^=14.97, p=0.002). The mean survival in patients with tumours that did not express CYP8B1 (n=201) was 128 months (95% CI 113-141), declining to 119 months (95% CI 104-134) for tumours with weak CYP8B1 immunostaining (n=171). The mean survival for CYP8B1 moderately expressing tumours (n=133) was 101 months (95% CI 89-113) and the mean survival for CYP8B1 strongly expressing tumours (n=118) was 79 months (95% CI 67-91). When negative/weak staining for CYP8B1 was compared to moderate/strong staining, there was a statistically significant relationship between CYP8B1 expression and survival (HR=1.376, 95% CI=1.093-1.731, χ^2^=7.511, p=0.006). The mean survival in patients with tumours showing negative/weak CYP8B1 immunoreactivity (n=371) was 126 months (95% CI 115-136) compared to 91 months (95% CI 82-99) in patients with tumours showing moderate/strong immunoreactivity (n=251). Comparing CYP8B1 negative/weak/moderate tumours with CYP8B1 strongly expressing tumours demonstrated a highly significant relationship with survival (HR=1.649, 95% CI=1.268-22.145, χ^2^=14.298, p<0.001). Patients with strongly staining tumours for CYP8B1 (n=118) survived a mean of 79 months (95% CI 67-91) whereas negative/weak/moderate CYP8B1 immunostaining was associated with a better prognosis and a mean survival of 123 months (95% CI 114-132).

CYP27A1 expression was associated with patient outcome in three out of four comparisons. Considering each CYP27A1 stain intensity separately, expression was significantly associated with survival (HR=1.144, 95% CI=0.986-1.328, χ^2^=7.863, p=0.049). Patients with strongly scoring tumours for CYP27A1 (n=16) had decreased survival, with a mean survival of 59 months (95% CI 35-84) in this group. For CYP27A1 negative tumours (n=367) the mean survival was 118 months (95% CI 108-128), for CYP27A1 weakly stained tumours (n=195) the mean survival was 108 months (95% CI 96-120) and for CYP27A1 moderately stained (n=44) tumours the mean survival was 91 months (95% CI 71-111). When negative/weak CYP27A1 immunostaining was compared to moderate/strong immunostaining, higher intensities of staining were significantly associated with poorer survival (HR=1.425, 95% CI=1.006-2.02, χ^2^=4.038, p=0.044). The mean survival in patients with tumours showing moderate/strong staining for CYP27A1 (n=60) was 82 months (95% CI 65-98) whereas for negative/weak tumours (n=562) the mean survival was 119 months (95% CI 110-127). Investigating strong CYP27A1 immunostaining compared to all other stain intensities was also significantly associated with survival (HR=2.093, 95% CI=1.2-3.651, χ^2^=7.135, p=0.008). Strong CYP27A1 immunostaining (n=16) resulted in a mean survival of 59 months (95% CI 35-84) compared to 118 months (95% CI 109-126) for negative/weak/moderate staining for CYP27A1 (n=606).

Immunohistochemistry for CYP39A1 did not reveal any strong staining. In a comparison of negative versus weak versus moderate staining for CYP39A1, a highly statistically significant association with survival was identified (HR=1.533, 95% CI=1.237-1.898, χ^2^=25.144, p<0.001). Tumours showing negativity for CYP39A1 (n=453) were associated with a mean survival of 125 months (95% CI 115-134), for CYP39A1 weakly stained tumours (n=164) the mean survival was 89 months (95% CI 79-99) and for CYP39A1 moderately stained tumours (n=12) the mean survival was 33 months (95% CI 16-50). Comparing CYP39A1 negatively staining tumours versus CYP39A1 positively staining tumours, CYP39A1 expression demonstrated a statistically significant relationship with survival (HR=1.468, 95% CI=1.157-1.861, χ^2^=10.21, p=0.001). Patients whose tumours did not express CYP39A1 (n=453) had a mean survival of 125 months (95% CI 115-135) whereas patients with weak/moderate staining for CYP39A1 (n=176) had a mean survival of 86 months (95% CI 76-96). Comparing negative/weakly stained tumours for CYP39A1 with moderately stained tumours revealed a highly significant relationship between expression and survival (HR=3.514, 95% CI=1.917-6.440, χ^2^=18.974, p<0.001). The mean survival for CYP39A1 negative/weak staining tumours (n=617) was 117 months (95% CI 109-126), declining to 33 months (95% CI 17-50) for patients whose tumours demonstrated moderate immunostaining for CYP39A1 (n=12).

Overall expression of CYP46A1 considering negative, weak, moderate and string staining as separate groups showed a statistically significant relationship with survival (HR=1.151, 95% CI=1.021-1.296, χ^2^=8.515, p=0.036). Tumours that were negative for CYP46A1 (n=74) showed a mean patient survival of 117 months (95% CI 95-138), weakly stained tumours (n=173) had a mean survival of 128 months (95% CI 113-144) and moderately stained tumours (n=205) had a mean survival of 113 months (95% CI 100-126). Strong staining reflected a poorer outcome, with patients who had strongly staining tumours for CYP46A1 (n=173) surviving a mean of 87 months (95% CI 76-98). A comparison of strong CYP46A1 immunostaining versus all other stain intensities was also significantly associated with survival (HR=1.422, 95% CI=1.115-1.813, χ^2^=8.179, p=0.004). In common with all other associations noted, higher CYP46A1 expression was linked to poorer prognosis. Tumours demonstrating strong CYP46A1 immunostaining (n=173) had a mean patient survival of 87 months (95% CI 76-98) whereas negative/weak/moderate CYP46A1 immunostaining (n=452) was associated with a mean patient survival of 122 months (95% CI 112-131).

## DISCUSSION

Colorectal cancer is one of the commonest types of solid tumour worldwide with an incidence that is still increasing especially in specific geographic areas [1]. While the molecular pathways involved in the initiation and the early stages of the development of colorectal cancer have been well defined this type of tumour still has a relatively poor prognosis with an overall survival of about 50-60%. The introduction of screening programs for its earlier detection and the development of targeted therapies for locally advanced and metastatic disease should impact on and improve the outcome from this disease [2, 19]. However, there is still a clear requirement to identify biomarkers of colorectal cancer which can contribute to improved screening and earlier diagnosis and prognostic stratification [3, 20].

This study has identified the expression profile of oxysterol metabolising enzymes in a well-characterised uniform cohort of primary colorectal cancers none of which had received pre-operative chemotherapy and/or radiotherapy. The expression of each enzyme was studied in primary colorectal cancer, corresponding lymph node metastasis and normal colonic mucosa. An oxysterol metabolising enzyme expression profile or signature associated with prognosis was identified.

Oxysterol metabolising enzymes are members of the cytochrome P450 superfamily of enzymes which catalyse NADPH-dependent mono-oxygenation reactions [21]. The cytochromes P450s are generally considered to belong to one of two distinct groups depending on whether they metabolise xenobiotics or endogenous substances and are classified into families, subfamilies and individual forms according to sequence homology and substrate specificity [22–25]. The major xenobiotic metabolising cytochrome P450s are members of the CYP1, CYP2 and CYP3 families. There is extensive evidence for the expression of xenobiotic metabolising enzymes in tumours [26–29]. There was no evidence of intra-tumour heterogeneity and this is consistent with our previous studies of other cytochrome P450 enzymes in tumours [29, 30, 31]. With some cytochrome P450s especially CYP1B1 showing increased expression in tumour cells and the tumour associated expression of individual cytochrome P450s has been exploited as therapeutic targets for P450 mediated pro-drug activation and as a cancer vaccine [29, 32–34]. The cytochrome P450s involved in the metabolism of a diverse range of endogenous compounds including eicosanoids, fatty acids, steroids and vitamins are the CYP4 family and higher numbered cytochrome P450 families.

Oxysterols are derived from cholesterol and can be produced by the hydroxylation of cholesterol by specific cytochrome P450 enzymes [35, 36]. Tumour-derived oxysterols are multifunctional lipid-signalling molecules and recent evidence indicates that they have pleiotropic effects in tumours [9]. Individual oxysterols have been identified as having a variety of functions in tumours including influence on tumour cell proliferation and tumour growth, mediating the tumour microenvironment especially of immune cell function and inflammation, tumour invasion and metastasis via the matrix metalloproteinase system and also mediating tumour associated angiogenesis [7, 12, 14, 16, 37]. The overall influence of oxysterols on tumour biology will depend on the relative expression of individual oxysterol metabolising enzymes. Although this study has focused on oxysterol pathway and its influence in tumour progression and metastasis, it is worth noting that cytochrome P450 enzymes have pleotropic functions that might also impact on tumour progression by modulating other signalling pathways.

In this study monoclonal antibodies with specificity for individual oxysterol metabolising enzymes CYP2R1, CYP7B1, CYP8B1, CYP27A1, CYP39A1, CYP46A1 and CYP51A1, have been produced. Peptides to C-terminal amino acid sequences identified on the basis of sequence alignment and homology modelling of individual cytochrome P450s were used as immunogen as this approach has proved highly successful in the development of monoclonal antibodies selective for individual cytochrome P450s [26].

CYP2R1, CYP7B1, CYP8B1, CYP46A1 and CYP51A1 all showed significantly increased expression in primary colorectal cancer compared to normal colonic mucosa with CY7B1 demonstrating the highest proportion of strong immunoreactivity in colorectal cancer compared to all other enzymes studied. This is the first study to analyse the expression of CYP7B1 in colorectal cancer, with previous research focusing on expression levels in prostate and breast cancer due to the role of CYP7B1 in sex hormone metabolism [38, 39]. CYP7B1 has been shown to be associated with survival in both breast cancer and prostate cancer [36, 37]. The findings of increased expression of CYP8B1 and CYP46A1 in primary colorectal cancer are novel findings. This study found increased expression of CYP51A1 in primary colorectal cancer compared with normal colonic mucosa. This finding is consistent with a previous study that also found increased expression of CYP51A1 in primary colorectal cancer [28].

Hierarchical cluster analysis allows the unbiased identification of groups of cases with similar expression profiles. Cluster analysis of the whole patient cohort identified five clusters which were mapped to two groups that were of prognostic significance; a good prognosis group which demonstrated low expression of CYP2R1, CYP8B1, CYP27A1, and CYP46A1 in comparison to their expression in the poor prognosis group. Similar prognostic potential of CYP27A1 and CYP7B1 in breast cancer has been reported [18, 38]. That research highlighted the important roles played by the oxysterol metabolite 27-hydroxycholesterol which results from both anabolism and catabolism of CYP27A1 and CYP7B1 enzymes respectively in tumour pathophysiology. The fact that our study has examined all the key enzymes of oxysterol pathway in colorectal cancer will lead to a comprehensive understanding of roles played by such enzymes and their oxysterol metabolites in tumour. Multi-variate analysis confirmed independent prognostic significance. Of particular interest was the highly significant prognosis in a model containing only information available at the time of biopsy diagnosis of colorectal cancer. This is important as the concept is emerging of treating more patients diagnosed with colorectal with neoadjuvant therapy followed by either observational follow-up or salvage surgery and it will be essential to have prognostic or risk-stratification biomarkers for this scenario in which only tumour biopsies are available for study at the time of initial treatment decisions [40].

The mismatch repair pathway is one of the major pathways of colorectal cancer development [41, 42]. Tumours that lack key mismatch repair proteins are classified as mismatch repair defective or deficient or unstable. Those patients with mismatch repair deficient tumours are already regarded as a distinct subgroup in selecting patients for adjuvant therapy and indicating prognosis, therefore the mismatch repair proficient group are of particular interest. For example, recent studies indicate that mismatch repair deficient tumours respond to immune checkpoint anti-programmed cell death 1 inhibitors in contrast to mismatch repair proficient tumours which showed no response [43]. This is due to the fact that mismatch repair deficient tumours have a high mutational load in coded proteins increasing the probability of recognition and elimination by the immune system. On the other hand, mismatch repair proficient tumours have low mutational load, proving effective in evading immune system. If this is the case then it is very plausible to speculate that most of existing and ongoing immunotherapies, including anti-CTLA4 and anti-CD20 will have limited therapeutic effect on mismatch repair proficient CRC patients. It is well-established that mismatch repair proficient tumours represent the majority of colorectal cancer patients and frequently has a worse prognosis compared to mismatch repair deficient tumours hence in most need of novel therapies. In the mismatch repair proficient group, a protein signature was identified that was associated with prognosis. This reflected the same relative expression of each cytochrome P450 as the whole patient cohort good prognosis group compared to the poor prognosis group. In the mismatch repair deficient group there was also a trend towards poorer prognosis.

This study also assessed the phenotypic expression of each P450 in both primary colorectal cancer and paired lymph node metastasis. When the primary tumours of lymph node positive cases (Dukes C, stage 3) were compared to the paired lymph node metastasis, expression of CYP2R1, CYP8B1, CYP39A1, CYP46A1 and CYP51A1 were each significantly reduced in the lymph node metastasis. This highlights the role of the tumour microenvironment in influencing the expression of the target proteins, a concept increasingly recognised in studies of the metastatic spread of malignancy [44–46]. The findings of this study have provided further evidence of the potential role of the tumour microenvironment in altering the phenotype of cancer cells. The spatial organization and hence interactions of individual types of immune cells within lymph nodes are distinct from the microenvironment of the primary tumour and thus contributes to a microenvironment that has a different structure and function to that of the primary tumour [47]. The variation in phenotype observed in metastasis compared to the primary malignancy also highlights the difficulty in effectively treating metastatic disease. Treatments for metastatic disease are often guided by assessment of the primary tumour which, as confirmed by this study, does not necessarily reflect phenotypic expression of disease at metastatic sites.

**Figure 6:**
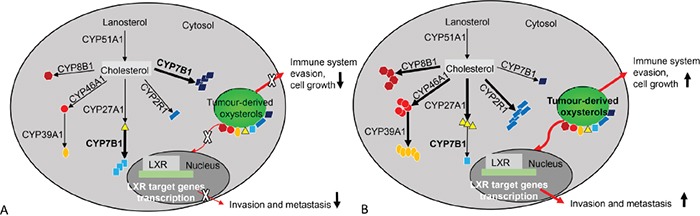
An overview of oxysterol metabolising enzymes in good and poor prognostic colorectal cancers **A.** Good prognosis colorectal cancers. CYP51A1, CYP27A1, CYP2R1, CYP46A1, CYP39A1 and CYP8B1 expression profile is low. This results in a lower concentration of oxysterols. Low oxysterol levels have less impact on promoting tumour invasion, metastasis, tumour cell survival and growth leading to a better survival outcome. However, increased expression of CYP7B1 correlates with good prognosis and this has been observed in breast cancer and prostate cancer. **B.** Poor prognosis colorectal cancers. CYP51A1, CYP27A1, CYP2R1, CYP46A1, CYP39A1 and CYP8B1 expression is increased in comparison with good prognostic tumours. This results in a significant increase in oxysterols. Oxysterols promote invasion and metastasis through LRX target genes and facilitates tumour cell survival and growth through immune system evasion and chemo-attraction of proangiogenic neutrophils.

In conclusion this study has defined the expression of oxysterol metabolising P450s in a well-characterised cohort of colorectal cancers. An oxysterol metabolizing enzyme signature has been identified which is associated with prognosis in the whole patient cohort and the mismatch repair proficient cohort. This good prognosis group showed tumours with low expression of CYP2R1, CYP8B1, CYP27A1 and CYP46A1 relative to the poor prognosis group and a schematic model of the relationship of oxysterol metabolizing enzymes in good and poor prognosis colorectal cancers is outlined in Figure 6. This study also raises the possibility of therapeutic targeting of the oxysterol metabolising pathway as individual P450s are well-characterised actionable drug targets [48].

## MATERIALS AND METHODS

### Monoclonal antibodies

Monoclonal antibodies to individual oxysterol metabolising enzymes (CYP2R1, CYP7B1, CYP8B1, CYP27A1, CYP39A1, CYP46A1, and CYP51A1) were developed in collaboration with Vertebrate Antibodies Ltd (Aberdeen, UK) using synthetic peptides. Peptides within the protein sequences were selected from regions which were antigenic, exposed on the surface and unique to the target protein [26, 49–51]. The amino acid sequences and location on the proteins are indicated in Table 7. The peptides were obtained from Almac Sciences Ltd, (Edinburgh, UK) and conjugated individually to ovalbumin for the immunisations and to bovine serum albumin for the ELISA test [50, 52]. The immunisation of mice, production of hybridoma cells and ELISA screening were carried out essentially as described previously except that the antigen was given by subcutaneous route [47]. The hybridomas were cloned by limiting dilution until a single ELISA positive colony was grown in a 96 well plate. Individual cell lines were then grown at high cell density for the preparation of the antibody stock which was used subsequently for their characterisation by immunoblotting and immunohistochemistry. All the antibodies are now commercially available from Vertebrate Antibodies.

**Table 7:** Peptide sequences used as immunogens to generate monoclonal antibodies

Enzyme	Hybridoma clone	Peptide sequence	Amino acid location
CYP2R1	M26P6H1	QPYLICAERR	492–501
CYP7B1	M17P3F2	IQYPDSDVL	491–499
CYP8B1	M15P3B7	QPSHDVRFR	486– 494
CYP27A1	V29P4B8*B2	KVVLAPETGEL	498–508
CYP39A1	M30P6D6	CRIEYKQRI	461–469
CYP46A1	N8P6E4*H8	PVLCTLRPR	481–489
CYP51A1	N6P2H5*G8	NPVIRYKRRS	493–502

### Immunoblotting

Whole cell lysates from cells (human embryonic kidney cells-HEK 293) overexpressing CYP2R1, CYP7B1, CYP8B1, CYP27A1, CYP39A1, CYP46A1 and CYP51A1 respectively were used as positive controls for immunoblotting while lysates from cells containing vector only were used as negative controls. The cell lysates and their corresponding controls were obtained from (Novus Biologicals, Cambridge, UK). Cell lysates (5 μg protein/lane) were resolved by electrophoresis on NuPAGE 4-12% Bis-Tris gels (Fisher Scientific, Loughborough, UK). Following protein transfer to nitrocellulose membrane the membranes were washed for 45 minutes at room temperature in phosphate buffered saline-Tween-20 (PBST) containing 3 % (w/v) skimmed milk powder to block non-specific protein binding. Membranes were incubated overnight at 4°C with individual monoclonal antibodies diluted in PBST (1/2 dilution) and then washed 6 times for a total of 60 minutes in 1% skimmed milk. The membranes were subsequently probed for 60 minutes with a secondary antibody conjugated horseradish-peroxidase-conjugated anti-mouse IgG (1/2000, Sigma-Aldrich, Dorset, UK). Membranes were then washed (6 times) for a total of 60 minutes in 1% skimmed milk and protein bands visualized using the enhanced chemiluminescence detection system (Fisher Scientific) [26, 49].

### Colorectal cancer tissue microarray

The patient cohorts of mismatch repair proficient and deficient colorectal cancers included 650 patients with a histologically confirmed diagnosis of primary colorectal cancer. All patients had undergone elective surgery for primary colorectal cancer, at Aberdeen Royal Infirmary (Aberdeen, UK), between the years of 1994 and 2009. Any patients who had received neoadjuvant chemotherapy and/or radiotherapy were excluded. Tissue was obtained retrospectively from the Aberdeen Colorectal Tumour Bank the Grampian Biorepository (www.biorepository.nhsgrampian.org), Aberdeen, UK provides governance for this tissue bank, see ethics statement). Survival information, in the form of all-cause mortality (i.e. overall survival), was available for each patient. At the time of censoring patient outcome data there had been 309 (47.5%) deaths. The mean patient survival was 115 months (95% CI 108-123 months). The study was conducted according to REMARK criteria and clinico-pathological characteristics of the patients and their tumours and relationship with survival are detailed in Table 8 and Supplementary Table 1.

**Table 8:** Clinico-pathological characteristics of all patients and their tumours and the relationship of each variable with overall survival

Characteristic	Number of patients	Percentage	Relationship with overall survival
Sex			
Male	340	52.3	χ^2^= 0.027, p=0.870
Female	310	47.7	
Age			
<70	305	46.9	χ^2^=29.213, **p<0.001**
≥70	345	53.1	
Screen detected			
Yes	52	8	χ^2^=16.381, **p<0.001**
No	598	92	
Tumour site			
Proximal colon	261	40.2	Proximal v distal, χ^2^= 8.418, **p=0.004**
Distal colon	245	37.7	Distal v rectal, χ^2^= 0.906, p=0.341
Rectum	144	22.2	Colon v rectum, χ^2^=0.098, p=0.754
Tumour differentiation			
Well/moderate	600	92.3	χ^2^=0.976, p=0.323
Poor	50	7.7	
Extra mural venous invasion			
Present	140	21.5	χ^2^=100.946, **p<0.001**
Absent	510	78.5	
Mismatch repair protein status			
Defective	96	15.2	χ^2^=2.848, p=0.091
Proficient	536	84.8	
Tumour (pT) stage			
pT1	30	4.6	T1 v T2, χ^2^=0.382, p=0.536
pT2	114	17.5	T2 v T3, χ^2^=24.739, **p<0.001**
pT3	411	63.2	T3 v T4, χ^2^=30.159, **p<0.001**
pT4	95	14.6	
Lymph node (pN) stage			
pN0	364	56	N0 v N1, χ^2^=54.071, **p<0.001**
pN1	177	27.2	N1 v N2, χ^2^=17.636, **p<0.001**
pN2	109	16.8	
Dukes stage			
A	120	18.5	A v B, χ^2^=5.059, **p=0.025**
B	244	37.5	B v C, χ^2^=65.510, **p<0.001**
C	286	44	

Significant values are highlighted in bold.

The histopathological reporting of the tumours was conducted in line with The Royal College of Pathologists UK guidelines for the histopathological reporting of colorectal cancer resection specimens and incorporating guidance from TNM5 [53]. The histopathological processing of the colorectal cancer excision specimens is detailed in Supplementary information Materials and Methods S1.

A tissue microarray was constructed as described from blocks of formalin fixed, paraffin embedded tissue specimens and included 650 primary colorectal cancers, 285 lymph node metastasis and 50 samples of morphologically normal colonic mucosa obtained from resection specimens at a site at least 10cm distant from the tumour [26, 54, 55]. Two cores each measuring 1mm in diameter were examined per primary tumour, lymph node and normal tissue sample as detailed in the Supplementary information Materials and Methods S1.

### Immunohistochemistry

Immunohistochemistry for each antibody was carried out using the Dako EnVision™ system (Dako, Ely, UK) using a Dako autostainer as previously described [26, 49, 54]. Sections were soaked in xylene to remove paraffin then rehydrated in alcohol prior to immunohistochemistry. When required (CYP7B1, CYP27A1, CYP39A1, CYP46A1 and CYP51A1), antigen retrieval was performed by microwave oven heating in a citrate buffer solution. Slides were fully immersed in pre-heated citrate buffer (pH 6) then heated in an 800W microwave at full power for 20 minutes. The slides were then allowed to cool at room temperature and placed in cold running water to complete the cooling process. Antigen retrieval was not required for monoclonal antibodies to CYP2R1 and CYP8B1.

The initial step in the automated staining protocol was a wash buffer rinse (Dako). Next, slides were incubated with the primary antibody for 60 minutes then washed with buffer. Each primary antibody was applied as undiluted tissue culture supernatant. Slides were then washed in buffer and peroxidase enzyme block was applied for a period of 7 minutes and slides were then again rinsed with wash buffer. Subsequently, the pre-diluted peroxidase labelled polymer coupled to goat anti-mouse/rabbit secondary antibody was applied for 30 minutes then rinsed with buffer to remove any unbound antibody. The diaminobenzidine (DAB) substrate was then applied for 7 minutes to demonstrate sites of peroxidase activity before a final wash with buffer then water. Slides were immersed in 0.5% copper sulphate for 2 minutes to intensify the DAB stain then washed with running water. Finally, slides were immersed in filtered Harris haematoxylin to lightly counterstain the nuclei before being dehydrated in alcohol and xylene and mounted. Omitting the primary monoclonal antibody from the immunohistochemical procedure and replacing it with antibody diluent (Dako) acted as a negative control. Normal liver was used as a positive control for CYP2R1, CYP7B1, CYP8B1, CYP39A1, CYP46A1 and CYP51A1. Grade 3 breast cancer of no special type was used a positive control for CYP27A1.

Immunohistochemistry was also performed on whole sections of a sub-set of tumours to investigate possible intra-tumour heterogeneity. Sections from the same tissue blocks from which tissue cores were obtained were used.

Following the completion of the immunohistochemistry protocol, the slides were examined by light microscopy using an Olympus BX 51 light microscope (Olympus, Southend-on-Sea, Essex, UK) equipped with an Olympus C4040 camera (Olympus). The intensity of immunostaining was quantified using a semi-quantitative scoring method as previously described [26, 49, 54]. The intensity of immunostaining (negative, weak, moderate, strong) and its localisation (cytoplasmic, nuclear, membranous) was assessed in the first instance by one investigator (RS). Following this primary scoring, a second investigator (GIM) independently scored each pair of cores. In the case of any discrepancies (less than 5 % of cases, kappa=0.931), both investigators simultaneously re-assessed the core in order to reach an agreed score. The highest scoring core for each individual tissue sample was recorded.

### Assessment of mismatch repair protein status

Mismatch repair protein status had previously been assessed by immunohistochemistry using antibodies to MLH1 and MSH2 [26]. Mismatch repair protein status was recorded as either proficient or defective.

### Statistics

Statistical analysis of the data including the Mann-Whitney U test, Wilcoxon signed rank test, chi-squared test, Kaplan-Meier survival analysis, log-rank test and Cox multi-variate analysis (variables entered as categorical variables) including the calculation of hazard ratios and 95% confidence intervals was performed using IBM SPSS version 22 for Windows 7™ (IBM, Portsmouth, UK). The log rank test was used to determine survival differences between individual groups. A probability value of p≤0.05 was regarded as significant. The influence of different cut-off points in relation to survival was investigated by dichotomising the immunohistochemistry intensity score for each marker. The groups that were analysed were negative staining versus any positive staining, negative and weak staining versus moderate and strong staining and negative, weak and moderate staining versus strong staining.

Unsupervised hierarchical cluster analysis was carried out using the within-group average linkage method with Pearson correlation as the cluster measure and cluster analysis was performed without any transformation of the data or imputation of missing values.

### Ethics

The colorectal cancer tissue microarray is held under the auspices of the Grampian Biorepository which has delegated research ethics authority (11/NS/0015) from The North of Scotland research ethics committee to approve research projects involving human tissue and data. This project was approved by the Grampian Biorepository scientific access group committee (Tissue request No. 0002). Written consent for the formalin fixed wax embedded tissue samples included in the colorectal cancer tissue microarray was not required.

## SUPPLEMENTARY MATERIALS AND METHODS




