# Supplementary material for: Characterisation of the oxysterol metabolising enzyme pathway in mismatch repair proficient and deficient colorectal cancer
Source: Oncotarget. 2016 Jun 22;7(29):46509–27. doi: 10.18632/oncotarget.10224 (PMC5216813; doi:10.18632/oncotarget.10224)
Supplement: Supplementary file 1 [file oncotarget-07-46509-s001.pdf]

Characterisation of the oxysterol metabolising enzyme pathway in mismatch repair proficient and deficient colorectal cancer

SUPPLEMENTARY MATERIALS AND METHODS

Histopathological processing of colorectal cancer

The colorectal cancer excision specimens were received fresh in the diagnostic histopathology laboratory, opened along the anti-mesenteric border proximal and when appropriate distal to the tumour, washed in cold water and then fixed in 10% neutral buffered formalin for at least 48 hours at room temperature prior to further dissection and block selection. Representative tissue blocks were embedded in wax, sections were then stained with haematoxylin and eosin for histopathological diagnosis and when required tumour sections were also stained with elastic haematoxylin and eosin to permit further assessment of extramural venous invasion (EMVI). The tumours were reported according to The Royal College of Pathologists UK guidelines for the histopathological reporting of colorectal cancer resection specimens and which incorporates guidance from version 5 of the TNM staging system. The mean lymph node yield for all tumours in this study was 14.29 lymph nodes per tumour and for node negative tumours the mean lymph node yield was 15.07 (lymph node yield refers to the total number of lymph nodes retrieved from each colorectal cancer resection specimen).

Construction of colorectal cancer tissue microarray

A colorectal cancer tissue microarray was constructed containing normal colon mucosal samples (n=50), primary (n=650) and metastatic colorectal cancer samples (n=285). 99 tumours were from the period 1994-1998, 198 tumours were from 1999-2003 and 353 tumours were from the period 2004-2009. The metastases were all from tumour involved lymph nodes of the Dukes C cases. Each normal mucosal sample was acquired from at least 10 cm distant from the tumour. All the cases were reviewed and areas of tissue to be sampled were first identified and marked on the appropriate haematoxylin and eosin stained slide by an expert consultant gastro-intestinal pathologist (GIM). Two 1mm cores were then taken from these areas of the corresponding wax embedded block using a Beecher Instruments tissue microarrayer (Sun Prairie, WI, USA) and placed in a recipient paraffin block.

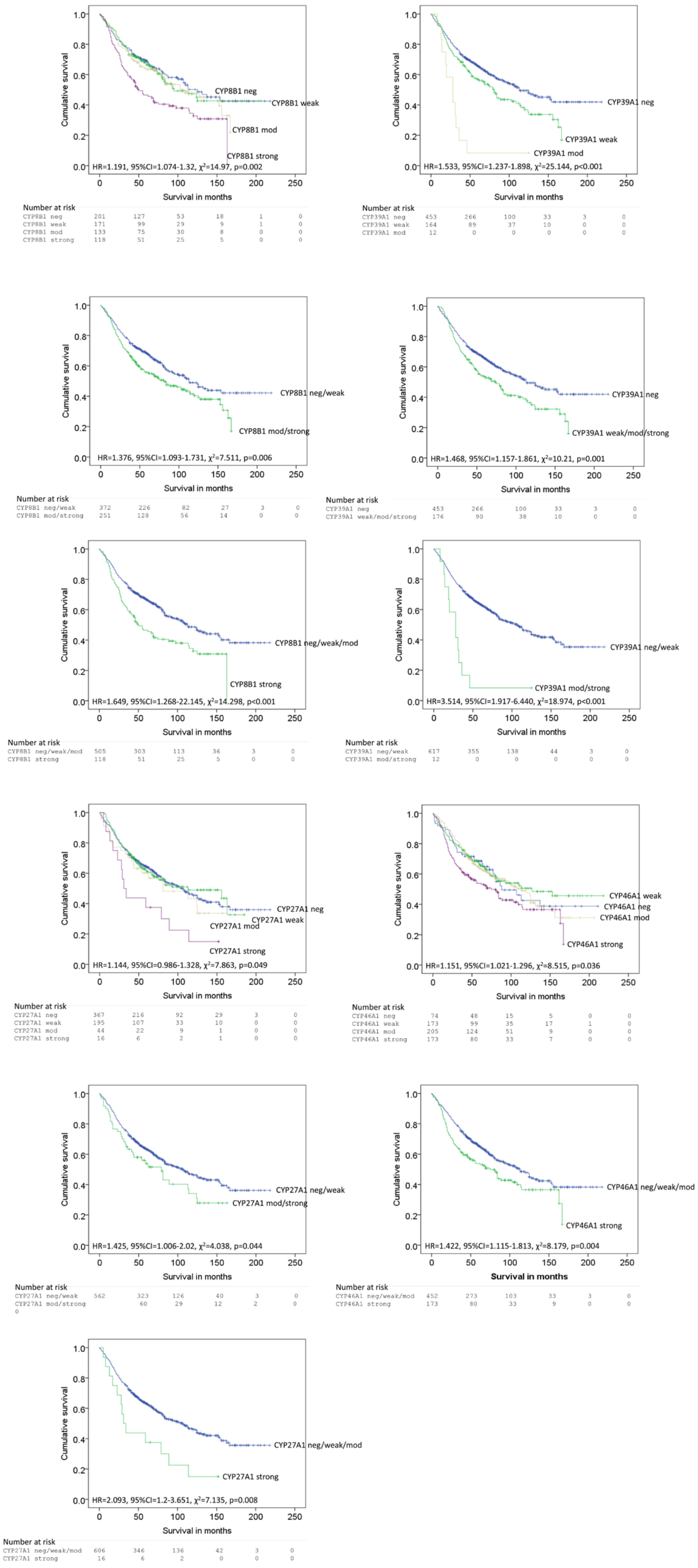

Supplementary Figure S1: The relationship of the expression of individual oxysterol metabolising enzymes and survival in the whole patient cohort.

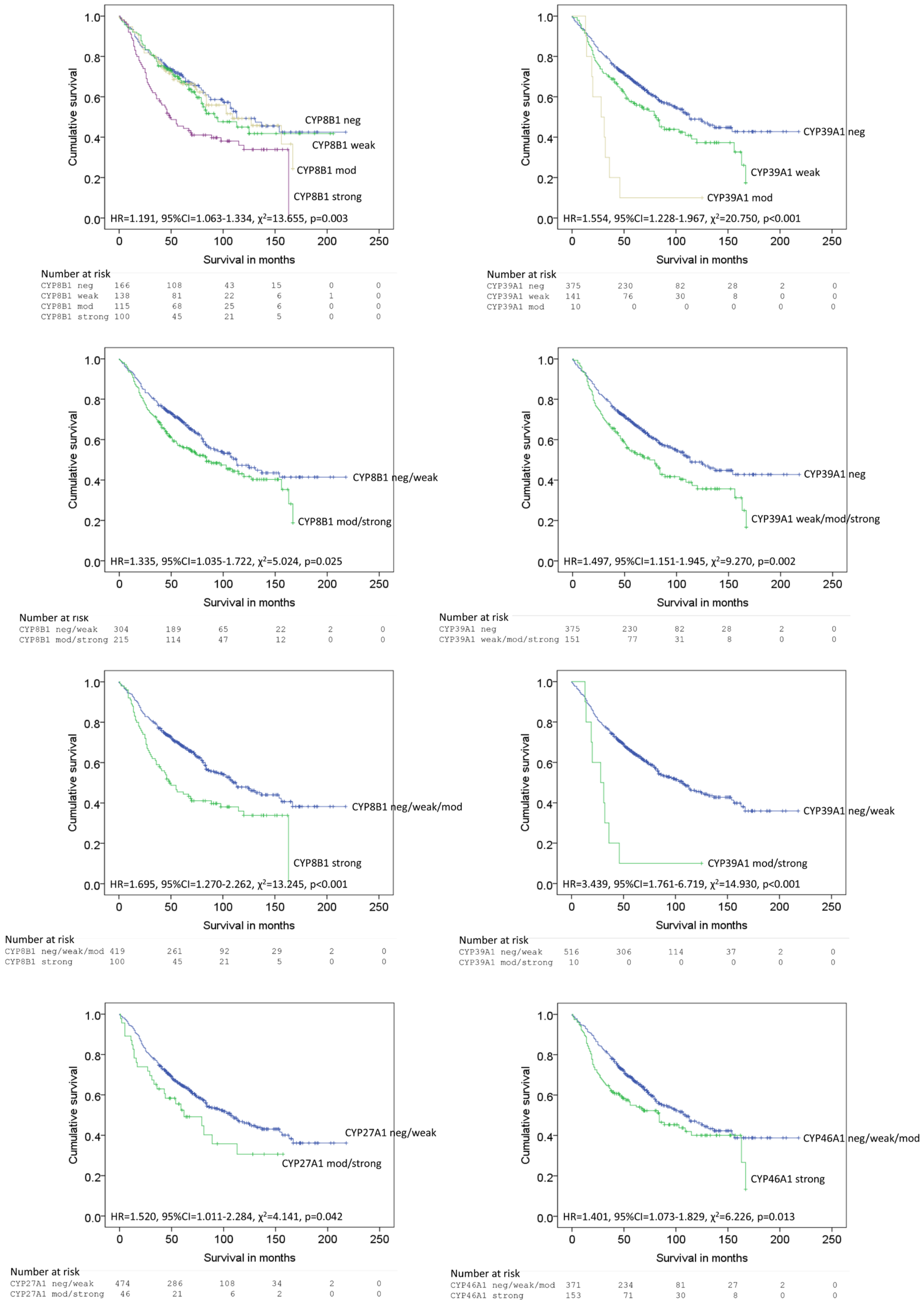

Supplementary Figure S2: The relationship of the expression of individual oxysterol metabolising enzymes and survival in patients with MMR proficient colorectal cancers.

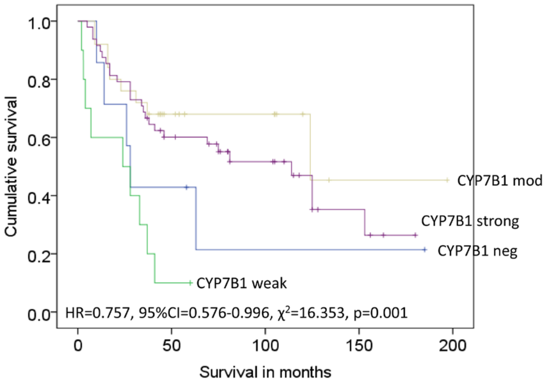

Number at risk

	0	50	100	150	200
CYP7B1 mod	25	10	6	0	0
CYP7B1 strong	48	25	13	3	0
CYP7B1 neg	7	2	0	0	0
CYP7B1 weak	10	0	0	0	0

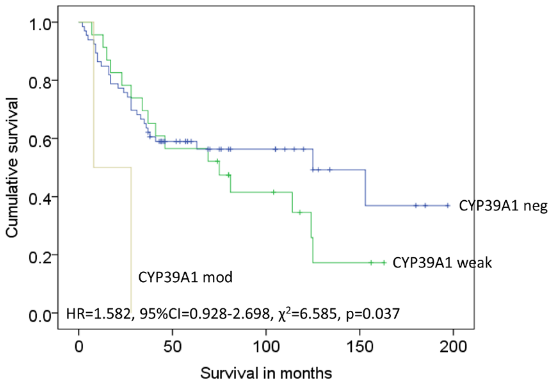

Number at risk

	0	50	100	150	200
CYP39A1 neg	66	29	13	3	0
CYP39A1 weak	23	12	6	1	0
CYP39A1 mod	2	0	0	0	0

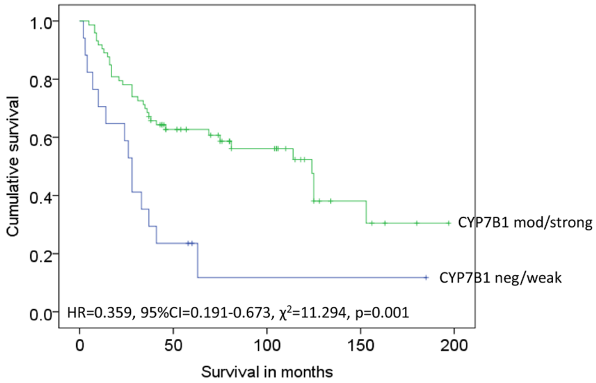

Number at risk

	0	50	100	150	200
CYP7B1 mod/strong	73	36	20	4	0
CYP7B1 neg/weak	17	3	0	0	0

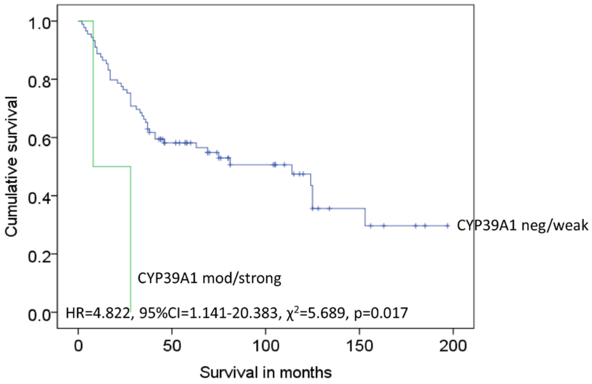

Number at risk

	0	50	100	150	200
CYP39A1 neg/weak	89	42	20	5	0
CYP39A1 mod/strong	2	0	0	0	0

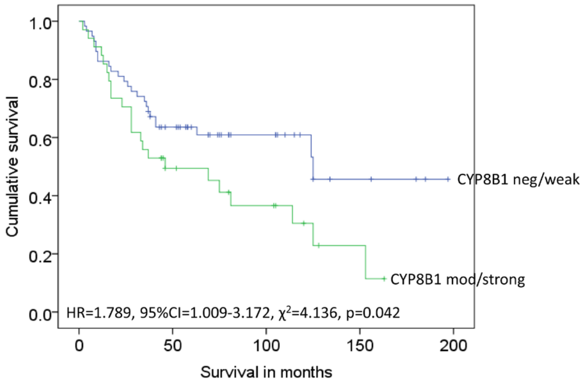

Number at risk

	0	50	100	150	200
CYP8B1 neg/weak	58	31	13	3	0
CYP8B1 mod/strong	34	12	7	1	0

Supplementary Figure S3: The relationship of the expression of individual oxysterol metabolising enzymes and survival in patients with MMR defective colorectal cancers.

Supplementary Table S1: Clinico-pathological characteristics of mismatch repair proficient and deficient cohorts

See Supplementary File 1

Supplementary Table S2: The relationship between expression of each oxysterol metabolising enzyme and pathological parameters

See Supplementary File 1

Supplementary Table S3: The relationship of the expression in the whole patient cohort of each oxysterol metabolising enzyme with survival (log rank test) using different cut-off points for the immunohistochemical intensity

	Negative versus weak versus moderate versus strong		Negative versus weak, moderate and strong		Negative and weak versus moderate and strong		Strong versus negative, weak and moderate	
	**χ2**	**p-value**	**χ2**	**p-value**	**χ2**	**p-value**	**χ2**	**p-value**
CYP2R1	4.803	0.187	1.920	0.166	1.672	0.196	0.533	0.465
CYP7B1	3.054	0.383	0.008	0.928	1.193	0.275	0.087	0.768
CYP8B1	14.970	**0.002**	3.631	0.057	7.511	**0.006**	14.298	**<0.001**
CYP27A1	7.863	**0.049**	0.421	0.517	4.038	**0.044**	7.135	**0.008**
CYP39A1	25.144	**<0.001**	10.210	**0.001**	18.974	**<0.001**	-	**-**
CYP46A1	8.515	**0.036**	0.224	0.636	3.097	0.078	8.179	**0.004**
CYP51A1	4.533	0.209	0.814	0.367	0.005	0.944	2.419	0.120

Significant values are highlighted in bold.

Supplementary Table S4: The relationship of the expression of oxysterol metabolising enzymes and survival in MMR proficient and defective colorectal cancers

See Supplementary File 1

Supplementary Table S5: The relationship between expression of each oxysterol metabolising enzyme and overall patient survival using individual cut-off points for immunostaining intensity with groups stratified by tumour site (colon v rectum)

See Supplementary File 1

Supplementary Table S6: The relationship of the expression of oxysterol metabolising enzyme and survival in proximal and distal colon cancers

See Supplementary File 1

Supplementary Table S7: The relationship of the expression of each oxysterol metabolising enzyme and survival in individual Dukes stage of colorectal cancer

See Supplementary File 1

Supplementary Table S8: The relationship of the expression of individual oxysterol metabolising enzymes and survival in colorectal cancers with and without EMVI

See Supplementary File 1

Supplementary Table S9: Multi-variate analysis of the whole patient cohort and mismatch repair proficient cohort including only parameters that would be available if cases are considered to be a biopsy i.e. no information regarding tumour stage, nodal stage or EMVI would be available

Variable	Whole patient cohort			Mismatch repair proficient cohort		
	Wald value	p-value	Hazard ratio (95% CI)	Wald value	p-value	Hazard ratio (95% CI)
Age (< 70 v ≥ 70)	24.814	**<0.001**	1.883 (1.468-2.461)	21.021	**<0.001**	1.896 (1.442-2.492)
Gender (M v F)	0.519	0.417	0.917 (0.725-1.160)	0.326	0.568	0.927 (7.14-1.203)
Tumour site (colon v rectum)	0.009	0.924	0.986 (0.745-1.306)	0.035	0.851	0.972 (0.719-1.313)
Tumour differentiation (well/ moderate v poor)	0.578	0.447	1.181 (0.769-1.813)	1.460	0.227	0.684 (0.369-1.267)
Cluster group (group 1 v group 2)	9.496	**0.002**	1.791 (1.236-2.595)	6.449	**0.011**	1.703 (1.129-2.568)

Significant values are highlighted in bold.
