# Supplementary material for: Characterisation of the oxysterol metabolising enzyme pathway in mismatch repair proficient and deficient colorectal cancer
Source: Oncotarget. 2016 Jun 22;7(29):46509–27. doi: 10.18632/oncotarget.10224 (PMC5216813; doi:10.18632/oncotarget.10224)
Supplement: Supplementary file 2 [file oncotarget-07-46509-s002.docx]

1

Table S1. Clinico-pathological characteristics of mismatch repair proficient and deficient cohorts

| Characteristic | Mismatch repair proficient cohort (n=536) | Percentage | Mismatch repair deficient cohort (n=96) | Percentage |
| --- | --- | --- | --- | --- |
| Sex |  |  |  |  |
| Male | 285 | 53.2 | 44 | 45.8 |
| Female | 251 | 46.8 | 52 | 54.2 |
| Age |  |  |  |  |
| <70 | 253 | 47.2 | 40 | 41.7 |
| ≥70 | 283 | 52.8 | 56 | 58.3 |
| Screen detected |  |  |  |  |
| Yes | 45 | 91.6 | 5 | 5.2 |
| No | 491 | 8.4 | 91 | 94.8 |
| Tumour site |  |  |  |  |
| Proximal colon | 184 | 34.3 | 67 | 69.8 |
| Distal colon | 226 | 42.2 | 17 | 17.7 |
| Rectum | 126 | 23.5 | 12 | 12.5 |
| Tumour differentiation |  |  |  |  |
| Well/moderate | 505 | 94.2 | 81 | 84.4 |
| Poor | 31 | 5.8 | 15 | 15.6 |
| Extra-mural venous invasion |  |  |  |  |
| Present | 110 | 20.5 | 27 | 28.1 |
| Absent | 426 | 79.5 | 69 | 71.9 |
| pT stage |  |  |  |  |
| T1 | 29 | 5.4 | 1 | 1 |
| T2 | 104 | 19.4 | 6 | 6.3 |
| T3 | 336 | 62.7 | 64 | 66.7 |
| T4 | 67 | 12.5 | 26 | 26 |
| pN stage (TNM5) |  |  |  |  |
| N0 | 317 | 59.1 | 40 | 41.7 |
| N1 | 138 | 25.7 | 29 | 30.2 |
| N2 | 81 | 15.1 | 27 | 28.1 |
| Dukes stage |  |  |  |  |
| A | 111 | 20.7 | 5 | 5.2 |
| B | 206 | 38.4 | 35 | 36.5 |
| C | 219 | 40.9 | 56 | 58.3 |

Table S2. The relationship between expression of each oxysterol metabolising enzyme and pathological parameters

|  | Screen detected | | Colon v rectum | | Tumour site | | Tumour differentiatio n | | EMVI | | MMR status | | Tumour stage | | Nodal stage | | Dukes stage | |
| --- | --- | --- | --- | --- | --- | --- | --- | --- | --- | --- | --- | --- | --- | --- | --- | --- | --- | --- |
|  | χ2 | p- value | χ2 | p-value | χ2 | p- value | χ2 | p- valu e | χ2 | p- value | χ2 | p- value | χ2 | p- value | χ2 | p- value | χ2 | p- value |
| CYP2R1 | 1.564 | 0.668 | 0.690 | 0.876 | 10.94 | 0.090 | 3.492 | 0.32 | 2.186 | 0.535 | 5.296 | 0.151 | 21.22 | **0.012** | 10.06 | 0.122 | 13.14 | **0.041** |
|  |  |  |  |  | 0 |  |  | 2 |  |  |  |  | 5 |  | 1 |  | 2 |  |
| CYP7B1 | 4.147 | 0.246 | 8.004 | **0.046** | 8.515 | 0.203 | 3.231 | 0.35  7 | 0.305 | 0.959 | 5.446 | 0.142 | 5.241 | 0.813 | 8.267 | 0.219 | 7.359 | 0.289 |
| CYP8B1 | 1.111 | 0.774 | 19.71 | **<0.001** | 21.59 | **0.001** | 4.502 | 0.21 | 8.577 | **0.035** | 0.785 | 0.853 | 7.536 | 0.581 | 32.76 | **<0.00** | 29.84 | **<0.00** |
|  |  |  | 2 |  | 3 |  |  | 2 |  |  |  |  |  |  | 6 | **1** | 4 | **1** |
| CYP27A1 | 3.207 | 0.361 | 4.189 | 0.242 | 21.39 | **0.002** | 2.866 | 0.41 | 16.318 | **0.001** | 10.00 | **0.018** | 14.08 | 0.119 | 11.04 | 0.087 | 13.55 | **0.035** |
|  |  |  |  |  | 4 |  |  | 3 |  |  | 9 |  | 5 |  | 8 |  | 5 |  |
| CYP39A1 | 12.651 | **0.002** | 7.851 | **0.020** | 8.357 | 0.079 | 0.271 | 0.87 | 12.643 | **0.002** | 0.120 | 0.942 | 16.76 | **0.010** | 39.19 | **<0.00** | 32.46 | **<0.00** |
|  |  |  |  |  |  |  |  | 3 |  |  |  |  | 6 |  | 3 | **1** | 3 | **1** |
| CYP46A1 | 2.139 | 0.544 | 8.657 | **0.034** | 12.30 | 0.055 | 1.378 | 0.71 | 7.964 | **0.047** | 4.664 | 0.198 | 19.28 | **0.023** | 16.70 | **0.010** | 29.30 | **<0.00** |
|  |  |  |  |  | 8 |  |  | 1 |  |  |  |  | 8 |  | 7 |  | 4 | **1** |
| CYP51A1 | 5.565 | 0.135 | 14.04 | **0.003** | 16.60 | **0.011** | 8.678 | **0.03** | 1.282 | 0.733 | 9.751 | **0.021** | 20.43 | **0.015** | 9.702 | 0.138 | 7.795 | 0.254 |
|  |  |  | 3 |  | 4 |  |  | **4** |  |  |  |  | 3 |  |  |  |  |  |

Significant values are highlighted in bold.

Table S4. The relationship of the expression of oxysterol metabolising enzymes and survival in MMR proficient and defective colorectal cancers.

|  | Negative versus weak versus moderate versus strong |  | Negative versus weak, moderate and strong |  | Negative and weak versus moderate and strong |  | Strong versus negative, weak and moderate |  |
| --- | --- | --- | --- | --- | --- | --- | --- | --- |
|  | χ2 | p-value | χ2 | p-value | χ2 | p-value | χ2 | p-value |
| CYP2R1 |  |  |  |  |  |  |  |  |
| Defective | 2.712 | 0.438 | 0.170 | 0.680 | 1.768 | 0.184 | 0.106 | 0.744 |
| Proficient | 5.775 | 0.123 | 2.535 | 0.111 | 0.948 | 0.330 | 1.481 | 0.224 |
| CYP7B1 |  |  |  |  |  |  |  |  |
| Defective | 16.353 | **0.001** | 0.831 | 0.362 | 11.294 | **0.001** | 0.752 | 0.386 |
| Proficient | 1.365 | 0.714 | 0.413 | 0.520 | 0.243 | 0.622 | 1.132 | 0.287 |
| CYP8B1 |  |  |  |  |  |  |  |  |
| Defective | 5.343 | 0.148 | 0.074 | 0.786 | 4.136 | **0.042** | 2.860 | 0.091 |
| Proficient | 13.655 | **0.003** | 3.255 | 0.071 | 5.024 | **0.025** | 13.245 | **<0.001** |
| CYP27A1 |  |  |  |  |  |  |  |  |
| Defective | 8.535 | **0.036** | 0.014 | 0.905 | 0.662 | 0.416 | 7.954 | **0.005** |
| Proficient | 4.256 | 0.235 | 0.856 | 0.355 | 4.141 | **0.042** | 1.463 | 0.226 |
| CYP39A1 |  |  |  |  |  |  |  |  |
| Defective | 6.585 | **0.037** | 1.696 | 0.193 | 5.689 | **0.017** | - | - |
| Proficient | 20.750 | **<0.001** | 9.270 | **0.002** | 14.930 | **<0.001** | - | - |
| CYP46A1 |  |  |  |  |  |  |  |  |
| Defective | 5.448 | 0.142 | 0.699 | 0.403 | 0.696 | 0.404 | 3.718 | 0.054 |
| Proficient | 6.707 | 0.082 | 0.732 | 0.392 | 3.242 | 0.072 | 6.226 | **0.013** |
| CYP51A1 |  |  |  |  |  |  |  |  |
| Defective | 7.609 | 0.055 | 1.889 | 0.169 | 0.821 | 0.365 | 2.329 | 0.127 |
| Proficient | 3.106 | 0.376 | 0.004 | 0.949 | 0.038 | 0.846 | 1.934 | 0.164 |

Significant values are highlighted in bold

Table S5. The relationship between expression of each oxysterol metabolising enzyme and overall patient survival using individual cut-off points for immunostaining intensity with groups stratified by tumour site (colon v rectum).

|  | Negative versus weak versus moderate versus strong |  | Negative versus weak, moderate and strong |  | Negative and weak versus moderate and strong |  | Strong versus negative, weak and moderate |  |
| --- | --- | --- | --- | --- | --- | --- | --- | --- |
|  | χ2 | p-value | χ2 | p-value | χ2 | p-value | χ2 | p-value |
| CYP2R1 | | | | | | | | |
| Colon | 5.092 | 0.165 | 2.581 | 0.108 | 2.547 | 0.111 | 0.059 | 0.807 |
| Rectum | 1.297 | 0.730 | 0.007 | 0.935 | 0.031 | 0.860 | 1.205 | 0.272 |
| CYP7B1 | | | | | | | | |
| Colon | 1.103 | 0.776 | 0.097 | 0.755 | 0.213 | 0.644 | 0.075 | 0.784 |
| Rectum | 3.963 | 0.265 | 0.450 | 0.502 | 2.509 | 0.113 | 0.047 | 0.829 |
| CYP8B1 | | | | | | | | |
| Colon | 25.224 | **<0.001** | 4.185 | **0.041** | 10.731 | **0.001** | 24.761 | **<0.001** |
| Rectum | 0.188 | 0.979 | 0.075 | 0.784 | 0.002 | 0.961 | 0.134 | 0.715 |
| CYP27A1 | | | | | | | | |
| Colon | 13.968 | **0.003** | 1.155 | 0.283 | 5.357 | **0.021** | 11.059 | **0.001** |
| Rectum | 0.659 | 0.883 | 0.314 | 0.575 | 0 | 1.000 | 0.045 | 0.832 |
| CYP39A1 | | | | | | | | |
| Colon | 21.676 | **<0.001** | 12.899 | **<0.001** | 13.244 | **<0.001** | − | − |
| Rectum | 7.926 | **0.019** | 0.242 | 0.623 | 7.867 | **0.005** | − | − |
| CYP46A1 | | | | | | | | |
| Colon | 11.111 | **0.011** | 0.561 | 0.454 | 3.087 | 0.079 | 11.059 | **0.001** |
| Rectum | 1.057 | 0.788 | 0.401 | 0.527 | 0.277 | 0.599 | 0.045 | 0.832 |
| CYP51A1 | | | | | | | | |
| Colon | 3.512 | 0.319 | 0.322 | 0.570 | 0.035 | 0.851 | 2.439 | 0.118 |
| Rectum | 1.510 | 0.680 | 0.639 | 0.424 | 0.134 | 0.709 | 0.304 | 0.582 |

Significant values are highlighted in bold

Table S6. The relationship of the expression of oxysterol metabolising enzyme and survival in proximal and distal colon cancers.

|  | Negative versus weak versus moderate versus strong |  | Negative versus weak, moderate and strong |  | Negative and weak versus moderate and strong |  | Strong versus negative, weak and moderate |  |
| --- | --- | --- | --- | --- | --- | --- | --- | --- |
|  | χ2 | p-value | χ2 | p-value | χ2 | p-value | χ2 | p-value |
| CYP2R1 | | | | | | | | |
| Proximal | 2.419 | 0.490 | 1.067 | 0.302 | 1.053 | 0.305 | 0.155 | 0.693 |
| Distal | 0.761 | 0.859 | 0.564 | 0.453 | 0.222 | 0.637 | 0.004 | 0.952 |
| CYP7B1 | | | | | | | | |
| Proximal | 4.717 | 0.194 | 0.104 | 0.747 | 2.562 | 0.109 | 0.599 | 0.439 |
| Distal | 2.117 | 0.549 | <0.001 | 0.987 | 0.934 | 0.334 | 1.727 | 0.189 |
| CYP8B1 | | | | | | | | |
| Proximal | 24.248 | **<0.001** | 1.685 | 0.194 | 8.487 | **0.004** | 23.954 | **<0.001** |
| Distal | 4.742 | 0.192 | 1.977 | 0.160 | 2.515 | 0.113 | 4.200 | **0.040** |
| CYP27A1 | | | | | | | | |
| Proximal | 10.672 | **0.014** | 0.034 | 0.854 | 1.941 | 0.164 | 10.499 | **0.001** |
| Distal | 2.521 | 0.471 | 0.533 | 0.465 | 2.343 | 0.126 | 0.933 | 0.334 |
| CYP39A1 | | | | | | | | |
| Proximal | 16.567 | **<0.001** | 8.765 | **0.003** | 11.158 | **0.001** | - | - |
| Distal | 5.303 | 0.071 | 4.000 | **0.046** | 2.383 | 0.123 | - | - |
| CYP46A1 | | | | | | | | |
| Proximal | 4.805 | 0.187 | 0.243 | 0.622 | 0.219 | 0.640 | 3.844 | **0.050** |
| Distal | 7.682 | 0.053 | 1.726 | 0.189 | 3.813 | 0.051 | 6.685 | **0.010** |
| CYP51A1 | | | | | | | | |
| Proximal | 3.210 | 0.360 | 1.851 | 0.174 | 0.001 | 0.981 | 0.420 | 0.517 |
| Distal | 3.095 | 0.377 | 0.235 | 0.628 | 0.003 | 0.957 | 1.924 | 0.165 |

Significant values are highlighted in bold

Table S7. The relationship of the expression of each oxysterol metabolising enzyme and survival in individual Dukes stage of colorectal cancer.

| Dukes stage | Negative versus weak versus moderate versus strong |  | Negative versus weak, moderate and strong |  | Negative and weak versus moderate and strong |  | Strong versus negative, weak and moderate |  |
| --- | --- | --- | --- | --- | --- | --- | --- | --- |
|  | χ2 | p-value | χ2 | p-value | χ2 | p-value | χ2 | p-value |
| CYP2R1 | | | | | | | | |
| Dukes A | 1.964 | 0.580 | 0.762 | 0.383 | 0.221 | 0.638 | 0.640 | 0.424 |
| Dukes B | 0.089 | 0.993 | 0.076 | 0.783 | 0.023 | 0.879 | 0.000 | 0.982 |
| Dukes C | 2.656 | 0.448 | 0.371 | 0.542 | 1.299 | 0.254 | 0.035 | 0.852 |
| CYP7B1 | | | | | | | | |
| Dukes A | 0.920 | 0.821 | 0.169 | 0.681 | 0.029 | 0.865 | 0.629 | 0.428 |
| Dukes B | 3.786 | 0.286 | 0.286 | 0.593 | 0.138 | 0.711 | 2.575 | 0.109 |
| Dukes C | 2.423 | 0.489 | 0.038 | 0.846 | 1.779 | 0.182 | 0.033 | 0.855 |
| CYP8B1 | | | | | | | | |
| Dukes A | 2.289 | 0.515 | 0.485 | 0.486 | 0.131 | 0.718 | 1.655 | 0.198 |
| Dukes B | 0.900 | 0.825 | 0.130 | 0.719 | 0.757 | 0.384 | 0.483 | 0.487 |
| Dukes C | 1.870 | 0.600 | 0.521 | 0.471 | 0.773 | 0.379 | 1.805 | 0.179 |
| CYP27A1 | | | | | | | | |
| Dukes A | 3.309 | 0.191 | 0.268 | 0.605 | 2.010 | 0.156 | - | - |
| Dukes B | 6.499 | 0.090 | 0.330 | 0.566 | 4.444 | **0.035** | 1.104 | 0.293 |
| Dukes C | 1.623 | 0.654 | 1.071 | 0.301 | 1.094 | 0.296 | 0.837 | 0.360 |
| CYP39A1 | | | | | | | | |
| Dukes A | 0.146 | 0.703 | 0.146 | 0.703 | - | - | - | - |
| Dukes B | 0.081 | 0.777 | 0.081 | 0.777 | - | - | - | - |
| Dukes C | 4.682 | 0.096 | 1.702 | 0.192 | 3.938 | **0.047** | - | - |
| CYP46A1 | | | | | | | | |
| Dukes A | 1.494 | 0.684 | 0.357 | 0.550 | 0.268 | 0.605 | 0.395 | 0.530 |
| Dukes B | 1.417 | 0.702 | 0.003 | 0.955 | 0.980 | 0.322 | 0.002 | 0.962 |
| Dukes C | 7.006 | 0.072 | 1.909 | 0.167 | 0.093 | 0.760 | 3.670 | 0.055 |
| CYP51A1 | | | | | | | | |
| Dukes A | 1.610 | 0.657 | 0.674 | 0.412 | 0.760 | 0.383 | 0.108 | 0.742 |
| Dukes B | 0.483 | 0.923 | 0.085 | 0.770 | 0.031 | 0.860 | 0.410 | 0.522 |
| Dukes C | 4.179 | 0.243 | 1.114 | 0.291 | 0.947 | 0.330 | 0.683 | 0.409 |

Significant values are highlighted in bold

Table S8. The relationship of the expression of individual oxysterol metabolising enzymes and survival in colorectal cancers with and without EMVI.

| EMVI  status | Negative versus weak versus moderate versus strong |  | Negative versus weak, moderate and strong |  | Negative and weak versus moderate and strong |  | Strong versus negative, weak and moderate |  |
| --- | --- | --- | --- | --- | --- | --- | --- | --- |
|  | χ2 | p-value | χ2 | p-value | χ2 | p-value | χ2 | p-value |
| CYP2R1 | | | | | | | | |
| Present | 3.266 | 0.352 | 0.323 | 0.570 | 1.098 | 0.295 | 0.385 | 0.535 |
| Absent | 2.170 | 0.538 | 1.583 | 0.208 | 0.214 | 0.644 | 0.232 | 0.630 |
| CYP7B1 | | | | | | | | |
| Present | 11.371 | **0.010** | 1.650 | 0.199 | 2.028 | 0.154 | 0.687 | 0.407 |
| Absent | 2.787 | 0.426 | 0.572 | 0.449 | 1.013 | 0.314 | 0.242 | 0.623 |
| CYP8B1 | | | | | | | | |
| Present | 3.477 | 0.324 | 0.001 | 0.975 | 0.767 | 0.381 | 3.027 | 0.082 |
| Absent | 6.491 | 0.090 | 2.489 | 0.115 | 4.821 | **0.028** | 5.084 | **0.024** |
| CYP27A1 | | | | | | | | |
| Present | 2.500 | 0.475 | 0.186 | 0.666 | 1.121 | 0.290 | 0.157 | 0.692 |
| Absent | 1.654 | 0.647 | 0.493 | 0.482 | 1.036 | 0.309 | 1.080 | 0.299 |
| CYP39A1 | | | | | | | | |
| Present | 0.496 | 0.780 | 0.269 | 0.604 | 0.364 | 0.546 | − | − |
| Absent | 23.215 | **<0.001** | 5.542 | **0.019** | 19.802 | **<0.001** | − | − |
| CYP46A1 | | | | | | | | |
| Present | 3.971 | 0.265 | 1.712 | 0.191 | 0.032 | 0.858 | 1.611 | 0.204 |
| Absent | 2.800 | 0.424 | 0.010 | 0.922 | 1.075 | 0.300 | 2.509 | 0.113 |
| CYP51A1 | | | | | | | | |
| Present | 1.339 | 0.720 | 0.525 | 0.469 | 1.182 | 0.277 | 0.845 | 0.358 |
| Absent | 5.416 | 0.144 | 0.826 | 0.363 | 0.010 | 0.921 | 3.216 | 0.073 |

Significant values are highlighted in bold
